# New Insights into Prostate Cancer Susceptibility in European Caucasians: A Systematic Review and Meta-Analysis of CYP3A4 Pharmacogene

**DOI:** 10.3390/cancers18010058

**Published:** 2025-12-24

**Authors:** Maria Pagoni, Claudia Cava, George T. Tsangaris, Fotios Siannis, Nikolaos Drakoulis

**Affiliations:** 1 Research Group of Clinical Pharmacology and Pharmacogenomics, Faculty of Pharmacy, School of Health Sciences, National and Kapodistrian University of Athens, Panepistimiopolis Zografou, 15771 Athens, Greece; 2 Department of Science, Technology and Society, University School for Advanced Studies IUSS Pavia, 27100 Pavia, Italy; claudia.cava@iusspavia.it; 3 Proteomics Research Unit, Biomedical Research Foundation, Academy of Athens, 11527 Athens, Greece; gthtsangaris@bioacademy.gr; 4 Department of Mathematics, National and Kapodistrian University, 15784 Athens, Greece; fsiannis@math.uoa.gr

**Keywords:** *CYP3A4*, prostate cancer, SNPs, pharmacogene, European Caucasians, meta-analysis

## Abstract

Background/Objectives: Prostate cancer is the most frequent male malignancy. The incidence of disease varies among different ethnic groups. CYP3A polymorphisms are candidates for prostate cancer susceptibility studies. The aim of the present study is to investigate the ethnicity-related clinical impact of *CYP3A4* variants on prostate cancer risk. Methods: A systematic literature search and meta-analysis were conducted according to PRISMA guidelines. A total of 10 eligible studies, including 3116 prostate cancer cases and 3008 healthy controls, were analyzed. We evaluated the association between the CYP3A4*1B (rs2740574, −392 A > G) variant and prostate cancer risk in European Caucasians. Pooled odds ratios (ORs) with 95% confidence intervals (CIs) were calculated using six genetic models. Data were analyzed using fixed and random-effects models based on the I2 value of heterogeneity magnitude. Funnel plots and Egger’s linear regression tests were used to assess publication bias. Results: CYP3A4*1B was associated with prostate cancer susceptibility in the allele (G vs. A: OR = 1.32, CI = 0.91–1.93), dominant (AG + GG vs. AA OR = 1.41, CI = 0.95–2.09), recessive (GG vs. AA + AG, OR = 1.82, CI = 1.26–2.63), homozygous (GG vs. AA, OR = 1.92, CI = 1.32–2.77), heterozygous model (AG vs. AA, OR = 1.31, CI = 0.89–1.93) and co-dominant model (AG vs. AA + GG; OR = 1.27, CI = 0.88–1.85). Significant heterogeneity characterized the allele, as well as the dominant model (I2 = 84.1%, I2 = 80.0%). Egger’s tests (*p* < 0.05) and funnel plots did not identify publication bias. Conclusions: The present meta-analysis indicates that the G allele and GG genotype might affect prostate cancer susceptibility in European Caucasians; however, the validity and reliability of the results need to be examined in future research.

## Simple Summary

Prostate cancer is the most frequently diagnosed malignancy in men worldwide. The study aimed to assess the impact of CYP3A4*1B polymorphism on prostate cancer risk in populations of European Caucasian ancestry. Despite the heterogeneity observed in the allele and in the dominant model, I2 = 84.1% and I2 = 80.0%, respectively, the present meta-analysis of 10 studies, encompassing 3116 patients and 3008 healthy controls sourced from PubMed and Cochrane Library, reveals a significant association for the homozygous model (GG vs. AA, OR = 1.92, CI = 1.32–2.77) and the recessive (GG vs. AA + AG, OR = 1.82, CI = 1.26–2.63). Egger’s tests (*p* < 0.05) did not indicate a publication bias. These findings suggest a higher prostate cancer risk, especially for men who are carriers of the G allele. Further experimental data from genetic association studies are necessary to clarify the relationship between CYP3A4*1B (rs2740574, −392 A > G) polymorphism and prostate cancer susceptibility in European Caucasians.

## 1. Introduction

Prostate cancer is the most commonly diagnosed cancer in males worldwide, with GLOBOCAN 2022 reporting it among the top four most diagnosed cancers [1]. Epidemiological studies from 2000 to 2020 demonstrate that prostate cancer incidence is highest among Black men, followed by White, Hispanic, American Indian and Alaska Native (AIAN), and finally Asian men [2]. Notably, during 2007–2021, the incidence of distant-stage prostate cancer increased across all racial and ethnic groups, with a particularly pronounced rise in unstaged cases among Hispanic individuals [3]. Additional studies have documented an increase in prostate cancer between 2014 and 2020, while the COVID-19 lockdown contributed to a significant reduction in screening activities and a decrease in diagnoses in 2020 associated with limited access to healthcare services [4,5]. This trend has clinical relevance, as BPH is present in a substantial proportion of prostate cancer cases as it is reported that more than 50% of prostate cancer cases are associated with Benign Prostatic Hyperplasia (BPH) elsewhere in the gland [6,7] and are associated with about 3–20% of patients who had transurethral prostatectomy (TURP) or open prostatectomy for Benign Prostatic Hyperplasia that subsequently developed prostate cancer [7,8]. Results derived from meta-analytic and recent Mendelian randomization analysis support a substantial causal association between BPH and increased prostate cancer risk, reinforcing the clinical significance of monitoring patients with BPH, but the underlying biochemical mechanism is not yet fully understood [9,10,11].

The etiology of the disease encompasses several factors, including ancestry, age, nutrition, obesity, family history, genetic, epigenetic, and environmental factors, as well as cholesterol levels and metabolic syndrome [12,13,14,15,16,17,18,19,20,21,22,23,24,25]. It has been demonstrated that steroids, particularly specific levels of androgen hormones, affect the risk of developing prostate cancer [26,27,28,29]. Cholesterol, the sole precursor of steroids, regulates intratumoral androgenic signaling in prostate cancer, while alterations in cholesterol homeostasis have been shown to enhance key pathways associated with prostate cancer growth and aggressiveness [30,31]. Androgen’s activity is regulated through the androgen receptor (AR), which is a crucial player in tumor initiation and therapy resistance [32], and it is expressed in the prostatic epithelium and prostatic stroma cells [33,34,35,36,37,38]. Polymorphic variations in androgen-regulatory genes may contribute to an increased susceptibility [39], and recent GWAS studies confirm correlations of biosynthesis and metabolic pathway variants, such as *CYP17A1* and *CYP3A4*, with prostate cancer risk [40,41].

Testosterone has a key role in promoting prostate cell division. It is the main natural anabolic–androgenic steroid [42] and the principal male reproductive hormone produced essentially by the Leydig cells of the testis (95%) [43], and to a small extent (≈5%) by the peripheral conversion from the precursors dehydroepiandrosterone (DHEA) and androstenedione (4-dione) which are produced in the zona reticularis of adrenal glands [33]. Its biotransformation takes place in the liver [44]. Testosterone’s transport mechanism in prostate cells occurs via passive diffusion, which, in the case of tumor cells, is enhanced by facilitated diffusion [45,46].

Testosterone is converted by the action of 5-α-reductase type 2, which is encoded by the *SRD5A2* gene, to the more potent androgen, dihydrotestosterone (DHT), mainly in peripheral tissues [47,48]. Recent data indicate that stromal *SRD5A2* promotes prostate growth in Benign Prostatic Hyperplasia (BPH) via a paracrine *WNT5A-LEF1-IGF1* signaling axis. This finding highlights a mechanism through which androgen metabolism and stromal-epithelial interactions contribute to prostate enlargement and may predispose to neoplastic transformation [49,50,51]. Another fraction of testosterone and androstenedione (AD) is converted to estradiol (E2) and estrone (E1), through the catalytic action of the aromatase enzyme (CYP19A1) [52,53]. DHT interacts with the androgen receptor and promotes the expression of genes involved in the growth of the adult prostate. It has a significant role in amplifying the weaker hormonal signal of testosterone, although the unbalanced concentration might cause detrimental effects [54,55,56]. Compared to testosterone, dihydrotestosterone (DHT) is a more potent androgen and demonstrates a higher binding affinity for androgen receptors in prostate cells, which activation drives increased survival and cellular proliferation, thereby promoting benign hyperplasia (BPH) as well as carcinogenesis [57,58].

The major metabolic reactions of testosterone are hydroxylation and further oxidation through enzymatic intervention of CYP450 isoforms involved in the attachment of hydroxyl groups to the steroid ring [44]. In humans, the major hydroxylated metabolite of testosterone, predominantly formed by CYP3A4, is 6β-hydroxytestosterone (6β-OHT), which is experimentally used as a biomarker of hepatic CYP3A4 activity in both in vitro and in vivo studies [42]. However, for the formation of 6β-OHT metabolite other isoforms, CYP3A5, CYP3A7, CYP1A1/2, CYP2D6, CYP2C19 are also involved [44,59]. Among them, CYP3A5*3, the major member of the CYP450, apart from its significant role in exogenous carcinogens and the metabolism of drugs, is also implicated in the oxidation and inactivation of testosterone, and may therefore be associated with an increased risk of prostate cancer, particularly among African populations [60]. In addition, CYP3A4 catalyzes the formation of secondary and minor metabolites, namely 2β-OHT, 15β-OHT, 2α-OHT, and 11β-OHT, as illustrated by Pagoni et al. (2025) [61].

Testosterone and its hydroxylated metabolites are conjugated with sulfate or glucuronic acid and are eliminated in bile and urine, increasing the water solubility of the molecule and reducing or abolishing the activity of the androgen receptor. Thus, it has been considered that low levels or decreased CYP3A4 effectiveness might result in a minor capacity to deactivate testosterone, favoring its conversion to dihydrotestosterone (DHT), and increasing the risk of prostate hypertrophy and hyperplasia, especially over the age of 50, or incrementing prostate cancer development under conditions of intense DHT activity. Thereafter, dihydrotestosterone’s bioavailability is decreased by CYP3A4, which regulates the 2β-, 6β-, and 15β- hydroxylation of testosterone in the liver and prostate [42,61]. The *CYP3A4* gene [62,63] is a member of a cluster of cytochrome CYP450 genes located on band 7q21.1 of the human karyotype. It is the predominant isoform of CYP3A enzymes that metabolizes antidepressants, macrolide antibiotics, immunosuppressants, opioids, statins, and anticancer drugs [64]. Moreover, protein expression is induced by xenobiotics [65,66,67], glucocorticoids, and many drugs, including acetaminophen, codeine, cyclosporine A, diazepam, erythromycin, and chloroquine, but it also participates in the metabolism of steroids and carcinogenic compounds [68,69]. The combinatory effect of the polymorphic *CYP3A4* and those of other xenobiotic genes, such as *CYP17* and *GSTP1*, could impact oxidative stress, androgen availability, and the cellular response to environmental carcinogens, thereby modulating individual predisposition to prostate oncogenesis [70].

Although there is comparative histologic evidence of the decreased expression of CYP3A4 measured with staining immunoreactivity in prostate cancer subjects compared to 93% for benign epithelium, versus 75% of prostate tumors that express CYP3A4, there are many interindividual variations in CYP3A4 in the liver (>100-fold) and extra-hepatic tissues, depending on genetic factors [62]. The GnomAD database registered more than 856 different *CYP3A4* polymorphisms, of which more than one-third are missense exonic mutations modifying the protein structure [71]. Additionally, in the dbSNP database, although they have submitted 10252 records, only a minor portion of the genetic variants of the *CYP3A4* have been investigated in humans for their clinical significance [72]. This substantial variability accounts for the divergent drug responses observed among patients administered identical dosages of a CYP3A4 substrate. Several factors, including age, sex, hormonal profile, nutritional habits, lifestyle, smoking, use of supplements, and concomitant medications, have an impact on CYP3A4 activity [73].

In the present systematic review and meta-analysis, we aimed to assess and highlight the most important polymorphic pharmacogenetic markers of the *CYP3A4* gene associated with prostatic neoplasia, particularly with prostate cancer risk in European Caucasians.

## 2. Materials and Methods

### 2.1. CYPA3A4 SNP Reporting and Literature Search

Genomic variants at single base positions (SNPs) of *CYP3A4* were searched using SNPedia [74] and SNPdb [71,72]. A structured literature review of the PubMed database and the Cochrane Library was performed from August 1998 to April 2025 to identify clinical studies correlating *CYP3A4* genes utilizing all PGx polymorphisms, with evidence of prostate cancer risk. The search was performed using the following Boolean search term:

#### (CYP3A4) and (Prostate Cancer)

Articles in the English language, in human adult males aged over 18, were included. Initially, the abstracts and the full manuscripts were revised to decide eligibility. All eligible publications were retrieved, while their reference lists were examined for other relevant studies. The articles were independently assessed and were discussed between three authors (C.C., M.P., and N.D.) when there was uncertainty about eligibility.

### 2.2. Eligibility and Identification of Relevant Studies

The systematic review followed the recommendations of the Preferred Reporting Items for Systematic Reviews and Meta-Analyses (PRISMA) 2020 [75]. The protocol has not been registered. Studies included in this meta-analysis met the following inclusion criteria: (1) original cancer research, i.e., case–control studies or cohort studies; (2) studies involving European Caucasian ethnicity, in the case of studies including mixed ethnic populations, only data pertaining to the European Caucasian subgroup were extracted and analyzed; (3) the literature on the correlation between gene polymorphism and prostate cancer susceptibility using primary data; and (4) the allele and genotype distributions of CYP3A4*1B polymorphism in cases and controls described in detail.

During the screening phase, records were excluded if they were clearly irrelevant based on their titles and abstracts. Particularly, studies were excluded if they were unrelated to the study scope, were conducted on cell lines or on animal models, or were represented by secondary research sources, such as narrative or systematic reviews, meta-analyses, correspondence, or clinical trials unrelated to the research question.

At the eligibility stage, after full-text assessment, additional studies were excluded for the criteria that were defined as follows: (i) the absence of CYP3A4*1B genotyping data, (ii) studies with no appropriate comparison group (i.e., including only healthy subjects or only prostate cancer patients), (iii) studies not addressing prostate cancer, (iv) studies with overlapping populations, (v) studies including participants of ethnicities other than European Caucasian, and (vi) studies with insufficient methodological quality or inadequate data reporting.

Quality assessment was achieved through the following process: first, each eligible study was critically assessed independently by the reviewers for its methodological rigor, scrutinizing factors of study design, data collection methods, and potential sources of bias; second, the assessments were checked for consistency, and studies that did not meet quality criteria were excluded. The quality criteria were as follows: (1) the study clearly defined the *CYP3A4* polymorphisms examined; (2) the ethnicity or ethnicities of the subjects were clearly stated; (3) the study provided absolute or relative frequencies of A and G alleles and/or AA, AG and GG genotypes, separately for cases and controls; (4) case and control groups were sampled from the same source population; and (5) control group subjects were cancer- and BPH-free.

### 2.3. Data Extraction

The data extraction and screening of the literature search results were carried out according to the above inclusion and quality criteria. For each study, they were collected the following basic characteristics: first author’s name, publication year, country in which the study was carried out, ethnicity, sample size, source of cases and controls, SNPs, allele, and genotype frequencies, specifically, absolute frequencies of case and control subjects, number of A and G alleles, number of AA, AG, and GG genotypes. Data was collected into extraction sheets. A minimum number of subjects was not defined for individual studies to be included in this meta-analysis.

### 2.4. Statistical Analysis

The strength of the association between the CYP3A4*1B (A > G) polymorphism and prostate cancer risk was measured by odds ratios (ORs) with 95% confidence intervals (CIs), and was performed under several genetic models. The pooled ORs were estimated for the allele model (G vs. A), dominant model (AG + GG vs. AA), recessive model (GG vs. AG + AA), homozygous model (GG vs. AA), and heterozygous model (AG vs. AA). We also examined the AG vs. AA + GG codominant model.

Study variations and heterogeneities were examined using Cochran’s Q-statistic with *p* value < 0.05 as a cutoff for statistically significant heterogeneity [76,77]. We also quantified the effects of heterogeneity by using the I2 test (ranges from 0 to 100%), which represents the proportion of inter-study variability that can be attributed to heterogeneity rather than to chance [76,78], so the summary estimate was analyzed in a random-effects model (DerSimonian–Laird model). Otherwise, a fixed-effects model was applied (Mantel–Haenszel model) [79].

Meta-analysis Forest plots were produced and inspected for statistical heterogeneity. Categorization of heterogeneity was considered according to the Cochrane Handbook for Systematic Reviews of Intervention: 0–40% unimportant; 30–60% moderate; 50–90% substantial; 75–100% considerable heterogeneity [80]. The assessment of statistical significance in Egger’s tests (*p* < 0.05), as well as funnel plot symmetry, was evaluated to explore publication bias. All calculations required for the meta-analysis were performed using the R package, metafor (version 4.6–0) [81].

## 3. Results

### 3.1. Identification of CYP3A4 SNPs

For this meta-analysis, we selected 25 single-nucleotide polymorphisms of *CYP3A4* with clinical relevance. These are annotated in Table S1 and were identified through a literature search from 1995 to 2025. It was found that the most relevant variant that is predominantly studied in European Caucasians and is related to prostate tumors is CYP3A4*1B (rs2740574), alternatively termed *CYP3A4-V* [82,83,84,85].

### 3.2. Study Selection and Characteristics of Literature

**Figure 1:**
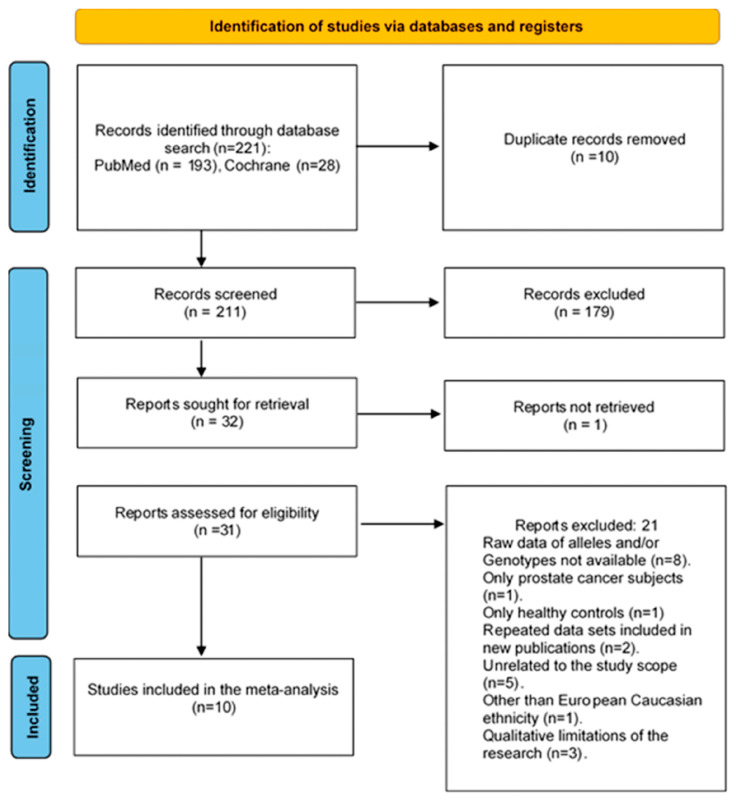
PRISMA 2020 flowchart outlining search strategy and the final list of included and excluded studies to verify the association of CYP3A4*1B polymorphism with prostate cancer risk in European Caucasians [75,86].

The initial electronic database search through literature identified 221 records for the period from 1998 to 2025. After removing 10 duplicates, 211 records were screened at the title and abstract level, and 179 were excluded. Evaluation of full-text review was performed on 31 of the remaining 32 articles, except for one study for which the full text could not be retrieved. In conclusion, 10 studies that met the eligibility criteria were included in the meta-analysis and 21 studies were excluded for the following reasons: raw data of alleles and/or genotypes were not available (n = 8 studies), only prostate cancer subjects (n = 1 study), only healthy controls (n = 1 study), repeated data sets included in new publications (n = 2 studies), unrelated to the study scope (n = 5 studies), other than European Caucasian ethnicity (n = 1 study), or qualitative limitations of the research (n = 3 study). Figure 1 shows the flow diagram of the selection process. 

The 10 eligible clinical studies [6,80,84,85,87,88,89,90,91,92] that were identified through the present meta-analyses describing the association of CYP3A4*1B with prostate cancer development are summarized in Table S2. They included data from 3116 prostate cancer patients and 3008 healthy controls, and were conducted from 1995 to 2025. These studies were conducted in European Caucasian populations and originated from different regions of the USA, UK, Brazil, South Africa, and Portugal (Table S2).

### 3.3. Quantitative Synthesis

**Figure 2:**
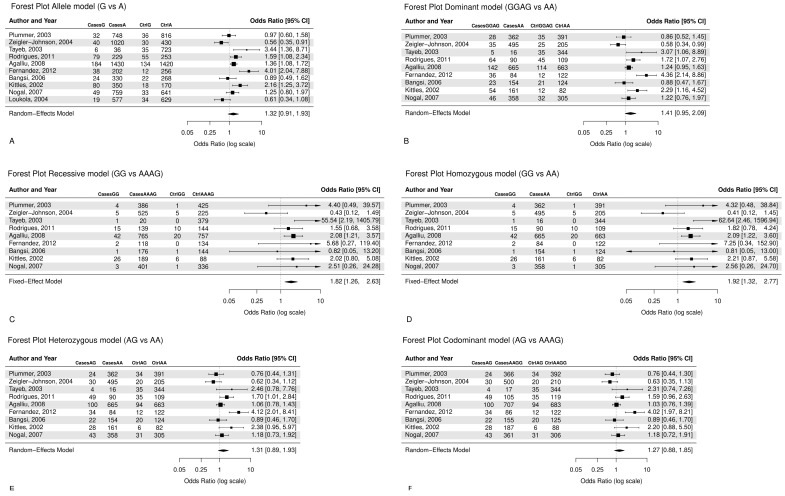
Forest plots with random and fixed effects meta-analysis refearred to [6,80,84,85,87,88,89,90,91,92] are reported for (**A**) allele model (G vs. A: OR = 1.32, CI = 0.91–1.93), (**B**) dominant model (AG + GG vs. AA: OR = 1.41, CI = 0.95–2.09), (**C**) recessive model (GG vs. AA + AG: OR = 1.82, CI = 1.26–2.63), (**D**) homozygous model (GG vs. AA, OR = 1.92, CI = 1.32–2.77), (**E**) heterozygous model (AG vs. AA, OR = 1.31, CI = 0.89–1.93), (**F**) codominant model (AG vs. AA + GG, OR = 1.27, CI = 0.88–1.85).

From all 10 studies that were pooled into the meta-analysis, although not significant, associations were found between CYP3A4*1B polymorphism and prostate cancer risk in the allele model (G vs. A: OR = 1.32, CI = 0.91–1.93), (Figure 2A), as well in the dominant model (GG + AG vs. AA, OR = 1.41, CI = 0.95–2.09), (Figure 2B), indicating that the G allele and GG/AG genotypes may increase the risk of prostate cancer. In addition, the recessive model (GG vs. AA + AG, OR = 1.82, CI = 1.26–2.63) (Figure 2C) presents a significant relationship between the GG genotype and prostate cancer risk, with cancer patients being 1.82 times more likely to have the GG genotype as opposed to the AA or AG genotypes. Similarly, the homozygous model (GG vs. AA, OR = 1.92, CI = 1.32–2.77) (Figure 2D) showed that the GG genotype was around two times more prevalent in cancer patients as opposed to the AA genotype. Moreover, the heterozygous model (AG vs. AA, OR = 1.31, CI = 0.89–1.93) (Figure 2E), studied under the random effect, indicates that the AG genotype has a 31% increased susceptibility to prostate cancer compared to the AA genotype. The codominant model (AG vs. AA + GG, OR = 1.27, CI = 0.88–1.85) (Figure 2F) suggests that the AG genotype is about 1.3 times more prevalent in cancer patients as opposed to the AA or GG genotype, though not significant.

### 3.4. Evaluation of Heterogeneity Among Studies

Table 1 and Table 2 show the heterogeneity and the pooled effect estimates for the six models. A significant association was found for the allele model (G vs. A, I2 = 84.1%, Q statistic *p* < 0.001), and the dominant model (GG + AG vs. AA, I2 = 80.0%, Q statistic *p* < 0.001). The recessive and homozygous models presented low heterogeneity (GG vs. AA + AG, I2 = 30%, *p* = 0.18) and (GG vs. AA, I2 = 34.0%, *p* = 0.15), respectively. The heterozygous model (AG vs. AA, I2 = 75.0% Q statistic *p* = 0.00) presented high heterogeneity. Moreover, the codominant model had high heterogeneity (AG vs. AA + GG, I2 = 73%, Q statistic *p* = 0.00). 

### 3.5. Publication Bias

**Figure 3:**
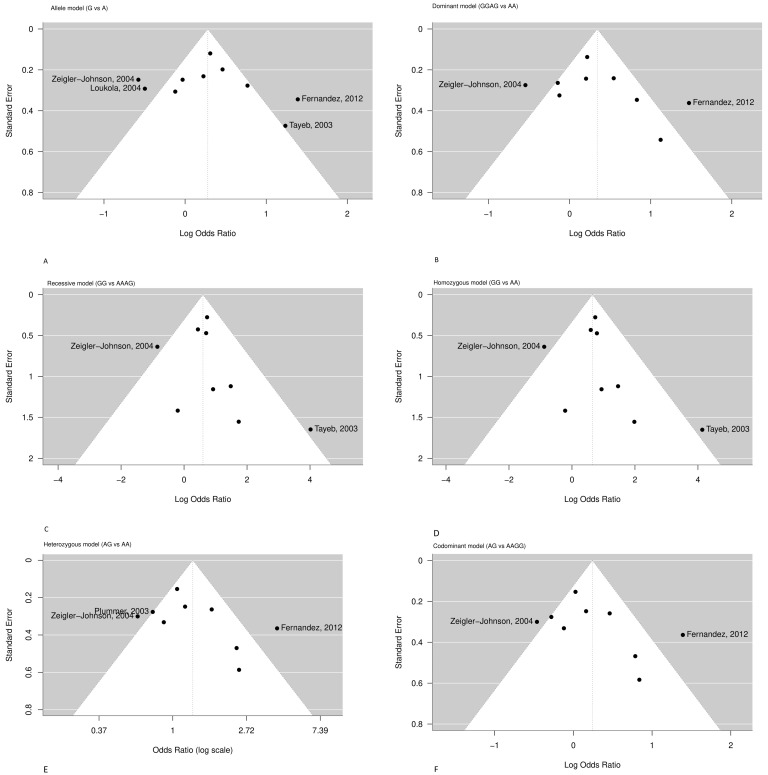
Funnel plots refearred to [6,80,84,85,87,88,89,90,91,92] assessing publication bias for the association between the CYP3A4*1B (rs2740574) polymorphism and prostate cancer risk under six genetic models: (**A**) Allele, (**B**) Dominant, (**C**) Recessive, (**D**) Homozygous, (**E**) Heterozygous, (**F**) Codominant.

Egger’s linear regression test did not suggest any significant publication bias in any genetic model (Table 3), although the number of studies was relatively small, comprising by maximum of 10 studies per model. The funnel plots depicted in Figure 3, for studies drawn in all genetic models, did not reveal any evidence of strong asymmetry for the polymorphism CYP3A4*1B studied in the meta-analysis.

The funnel plots are depicted in Figure 3. Most studies are symmetrically distributed within the expected range, suggesting the absence of major publication bias. However, mild asymmetry is visible in the allelic, recessive, and homozygous models, mainly driven by two outlier studies [6,88] ikely reflecting inter-study heterogeneity rather than publication bias.

## 4. Discussion

The human cytochrome P450 3A (*CYP3A*) subfamily of enzymes has an important role in metabolizing endogenous compounds and diverse xenobiotics. They assume extreme importance in the etiology of oncogenesis due to their contribution to the activation and degradation of carcinogens, steroids, as well as bioactivation of cytostatic prodrugs, particularly through their polymorphisms associated with prostate cancer in Caucasians and other ethnicities [41,61,93]. Within a region of 218 kb of chromosome 7q22.1 lie four *CYP3A* members of the cluster: *CYP3A5*, *CYP3A7*, *CYP3A4*, and *CYP3A43* [94,95]. In particular, the SNP rs2740574 variant, a single base change in the 5′-UTR, is reported to be located in the promoter region of the *CYP3A4* gene, in the nifedipine-specific response element. Numbering refers to the translation start site, and the base change affects the transcription efficiency, modifying the overall activity of the protein product [96,97]. The A→G promoter change (rs2740574, CYP3A4*1B) can regulate *CYP3A4* expression, but changed expression has been linked to modified steroid metabolism in prostatic tissue in particularly increased androgen bioavailability. This effect promotes androgen-mediated prostate carcinogenesis and altered metabolic activation of pro-carcinogens, enhancing cancer susceptibility [69,98,99,100].

The product of the variant is characterized by a substitution of Alanine for Glycine at codon 293 and has been associated with a 1.7 to 9.5-fold increase in the risk for prostate cancer [41,87]. The allele was first identified in 1998 by Rebbeck et al. [82], the same year that Walker et al. reported its comparative frequency of 9% among whites and 53% among African-Americans, and 0% in Taiwanese (Asians) [101]. Furthermore, *CYP3A4* has been shown to play a central role in both the activation and inactivation of anticancer drugs and other xenobiotics. This underscores the necessity of accounting for genotype-specific effects in prostate cancer management [102,103].

Although the CYP3A4*1B variant was initially reported to be associated with a higher clinical grade of prostate cancer, especially in patients over 65 with no family history [71,72,99,104], other studies found that this association might be attributed to population stratification, and not to prostate cancer susceptibility [82,97]. In pharmacokinetic (PK) studies, the variant was also associated with higher dose requirements of tacrolimus [96] and cyclosporine therapy in transplantations [105,106]. It was also related to a lower risk of dose decrease or changing treatment during simvastatin therapy [107]. Furthermore, it was observed that, when CYP3A4*1B, CYP3A5*3, and CYP3A4*22 were studied together, the enzymatic relevance of CYP3A4*1B was lower [108]. In addition, it was found that CYP3A4*1B is under significant linkage disequilibrium with CYP3A5*1 [71,106].

Consistent with these findings, recent population-based research has also revealed relationships between *CYP3A4* and *CYP3A5* genotypes and a higher risk of developing prostate cancer in a Bangladeshi population, linked to the presence of *1A/1B and *1B/1B genotypes of *CYP3A4* as well as to the *CYP3A5* gene’s *1/3 and *3/3 genotype [41]. Since *CYP3A5* is the major extrahepatic CYP3A isoform expressed in prostate tissue, which modulates androgen receptor (AR) activation, the influence on AR signaling is therefore primarily attributed to alterations in *CYP3A5* rather than *CYP3A4* [109,110].

CYP3A enzymes convert testosterone to a less active form, hydroxytestosterone, through hydroxylation in a regioselective and stereoselective fashion, thereby reducing AR activation in prostate cells. Conversely, the decreased expression of *CYP3A*, notably *CYP3A5*, restricts the catabolism of testosterone and increases AR activation, promoting oncogenesis. Although there is no evidence that the CYP3A4*1B polymorphism directly modifies androgen receptor (AR) expression or activation, its functional consequences on steroid metabolism may indirectly alter AR signaling. In particular, the reduced expression of CYP3A4 is associated with prostate cancer development and has an inverse correlation with Gleason score and patient prognosis. Furthermore, the CYP3A4*1B promoter variant is linked to higher tumor grade and advanced tumor stage due to ineffective androgen deactivation rather than direct regulation of AR expression [62,100].

Published data on the association between *CYP3A4* A392G and the risk of prostate cancer remains controversial. The discrepancy may be partially due to different gene–environmental interactions observed in previous studies of different cancer types [100,111]. To our knowledge, this is the first meta-analysis to address the associations between the CYP3A4*1B gene variant (rs2740574) and prostate cancer susceptibility in European Caucasian populations. Other meta-analyses performed on the reported literature investigating the association of the polymorphisms of *CYP3A4* and cancer risk [95,99,100] suggested that their frequency and their functions, particularly of the CYP3A4*1B polymorphism, were different among different ethnic groups, and the cancer susceptibility was variable [111]. Moreover, the findings of Zhou et al. [99] and He et al. [95] are similar to Zheng et al. [111], which also indicated that the CYP3A4*1B polymorphism might be associated with increased cancer risk.

This meta-analysis included 6232 allele records of prostate cancer cases and, respectively, 6015 controls from 10 original published studies that explored the association between a potentially functional polymorphism of *CYP3A4* and the susceptibility to the disease (Table S2). Specifically, it has been shown that prostate cancer patients were more likely to present the G allele over the A allele, with a G/A ratio of 551/5681 for prostate cancer patients and 409/5606 for healthy controls. In addition, in prostate cancer subjects, the AG and GG genotypes were detected in higher frequencies compared to healthy controls, i.e., for prostate cancer patients 334 (AG) and 99 (GG) times, and for healthy controls 287 (AG) and 44 (GG) times, respectively (Table S2).

The dominant model suggested a higher occurrence of the GG or AG genotypes among prostate cancer patients compared with controls, although this association was not statistically significant (Figure 2B). In contrast, both the recessive (Figure 2C) and homozygous (Figure 2D) models showed statistically significant associations, indicating that individuals carrying the GG genotype had approximately twice the risk of developing prostate cancer compared to those with the AA genotype or with AA/AG genotypes. However, the GG genotype was relatively rare (≈2% among controls), suggesting that this finding should be interpreted with caution due to limited statistical power.

Overall, our results are consistent with those reported by Zhou et al. [99], who found a similar trend, and extend them by including a specific ethnic and disease context. Indeed, Zhou et al. [99] performed the meta-analysis considering a variety of cancers across different ethnicities, whereas the present study focuses specifically on the association between CYP3A4*1B and prostate cancer susceptibility in European Caucasians. These additions strengthen the overall evidence that the CYP3A4*1B variant may modestly influence prostate cancer susceptibility in European Caucasians, although further large-scale studies are needed to confirm this effect.

Increased heterogeneity in the allele and dominant models introduces reduced precision of OR results, meaning that the extent to which the G allele and the GG/AG genotypes increase the prostate cancer risk could not be precisely estimated. Moreover, heterogeneity may indicate that the studies being combined in the meta-analysis are not directly comparable. Considering that meta-regression tests and funnel plots did not indicate publication bias, it may be argued that the source of heterogeneity could not be specified for the allele and dominant models; therefore, challenges are introduced for the generalization of the results to the population of European Caucasian males.

Therefore, from the data screening, it was observed that the study of Loukola et al. 2004 [91] provided evidence only for the A and G alleles’ frequency in prostate cancer patients and healthy controls, and it was only included in the allele model (Table S2) (Figure 2A). The study of Zeigler-Johnson et al. (2004) [88] in the allele model was an exception for the probability that the G allele would be present in prostate cancer patients at higher frequencies compared to healthy control cases (OR = 0.56, 95% CI = 0.35–0.91) (Figure 2A). Moreover, the increased heterogeneity of the allele model may be attributed to the four studies that deviate from the funnel plot area: Zeigler-Johnson et al. (2004), Loukola et al. (2004), Fernandez et al. (2012), and Tayeb et al. (2003) [6,85,88,91] (Figure 3).

Several limitations need to be acknowledged in the current meta-analysis. The present meta-analysis was intentionally restricted to European Caucasian populations to minimize population stratification and genetic heterogeneity, which could otherwise confound the association between *CYP3A4* polymorphisms and prostate cancer risk. Therefore, the limited generalizability of the findings to other ethnic groups (e.g., Asian or African populations) reflects this methodological choice rather than a limitation of sample size. Second, as a type of retrospective study, the present meta-analysis may have encountered selection bias, which can influence the reliability of our study results. In addition, previous studies in European Caucasians indicate that the CYP3A4*1B exists in linkage disequilibrium with functional variants of another *CYP3A* allele (CYP3A5*1) [89,93,112]. Therefore, the validity of the results needs to be verified in future research in broader European Caucasian cohorts.

Despite these limitations, our meta-analysis suggests that among all studied *CYP3A4* variants, CYP3A4*1B might play a role in susceptibility to prostate cancer in European Caucasians. However, it is essential to conduct large sample epidemiological studies using standardized, unbiased protocols and genotyping methods, homogeneous prostate cancer patients, and well-matched controls. Several additional studies about CYP3A4*1B on prostate cancer susceptibility would greatly improve the power of the present meta-analysis on this polymorphism.

## 5. Conclusions

In conclusion, our results indicate a potential association between CYP3A4*1B and prostate cancer predisposition, suggesting a role in the relationships of different genotypes in disease susceptibility.

## Figures and Tables

**Table 1:** Summary of heterogeneity statistics and model parameters for each genetic comparison.

	Allele (G vs. A)	Dominant (GGAG vs. AA)	Recessive (GG vs. AAAG)	Homozygous (GG vs. AA)	Heterozygous (AG vs. AA)	Codominant (AG vs. AAGG)
Model	RE model (k = 10)	RE model (k = 9)	FE model (k = 9)	FE model (k = 9)	RE model (k = 9)	RE model (k = 9)
tau^2^ (estimated amount of total heterogeneity)	0.2985 SE = 0.176	0.2758 SE = 0.1823	-	-	0.241 SE = 0.1716	0.2162 SE = 0.1582
tau (square root of estimated tau^2^ value)	0.5463	0.5251	-	-	0.4909	0.465
I^2^ (total heterogeneity/total variability)	84.08%	79.96%	29.91%	34%	74.84%	72.88%
H^2^ (total variability/sampling variability)	6.28	4.99	1.43	1.52	3.97	3.69
Q(df = 8), *p*-value	40.5448, <0.0001	30.3032, 0.0002	11.4143, 0.1793	12.1207, 0.1459	25.7157, 0.0012	24.1861, 0.0021

Note: SE: Standard Error.

**Table 2:** Pooled effect estimates (odds ratios) and 95% confidence intervals for each genetic model.

Model	Estimate	se	zval	pval	ci.lb	ci.ub
Allele (G vs. A)	0.2797	0.1936	1.445	0.1484	−0.0997	0.6592
Dominant (GGAG vs. AA)	0.3409	0.2021	1.6867	0.0917	−0.0552	0.7371
Recessive (GG vs. AAAG)	0.6003	0.1876	3.1997	0.0014	0.2326	0.9681
Homozygous (GG vs. AA)	0.6501	0.1886	3.4474	0.0006	0.2805	1.0198
Heterozygous (AG vs. AA)	0.2719	0.1966	1.3828	0.1667	−0.1135	0.6572
Codominant (AG vs. AAGG)	0.2427	0.1889	1.2844	0.199	−0.1276	0.613

**Table 3:** Results of Egger’s Regression-Based Test for all genetic models: allele, dominant, recessive, heterozygous, homozygous, and codominant genetic models.

Genetic Model	Egger’s Test *p*-Value *
Allele model (G vs. A)	0.80
Dominant model (GGAG vs. AA)	0.39
Recessive model (GG vs. AAAG)	0.53
Homozygous model (GG vs. AA)	0.52
Heterozygous model (AG vs. AA)	0.26
Codominant model (AG vs. AAGG)	0.25

* The number of studies is small (9 or 10 per model).

## Data Availability

All data and materials are publicly available.

## Supplementary Materials

The following supporting information can be downloaded at: https://www.mdpi.com/article/10.3390/cancers18010058/s1. References [113,114,115,116,117,118,119,120,121,122,123,124,125,126] are cited in the Supplementary Materials.
