# Supplementary material for: New Insights into Prostate Cancer Susceptibility in European Caucasians: A Systematic Review and Meta-Analysis of CYP3A4 Pharmacogene
Source: Cancers (Basel). 2025 Dec 24;18(1):58. doi: 10.3390/cancers18010058 (PMC12785120; doi:10.3390/cancers18010058)
Supplement: Supplementary file 1 [file cancers-18-00058-s001.zip › Table S1.pdf]

Table 1: Annotation of most frequent CYP3A4 variants reported from SNPedia.

Allele Name	Rs#	References
CYP3A4*1		[1,2]
CYP3A4*1B	rs2740574	[3-6]
CYP3A4*2	rs55785340	[7,8]
CYP3A4*3	rs4986910	[8,9]
CYP3A4*4	rs55951658	[10,11]
CYP3A4*5	rs55901263	[5,6]
CYP3A4*6	rs4646438	[5,6]
CYP3A4*8	rs72552799	[5,12]
CYP3A4*11	rs67784355	[5,12]
CYP3A4*12	rs12721629	[13]
CYP3A4*13	rs4986909	[13]
CYP3A4*14	rs12721634	[14]
CYP3A4*15A	rs4986907	[13]
CYP3A4*16	rs12721627	[14,15]
CYP3A4*17	rs4987161	[12]
CYP3A4*18	rs28371759	[15-17]
CYP3A4*19	rs4986913	[18]
CYP3A4*20	rs67666821	[13]
CYP3A4*_11460A>G(K96E)	rs3091339	[19]

References

1. Teichert, M.; Eijgelsheim, M.; Uitterlinden, A.G.; Buhre, P.N.; Hofman, A.; De Smet, P.A.; Visser, L.E.; Stricker, B.H. Dependency of phenprocoumon dosage on polymorphisms in the VKORC1, CYP2C9, and CYP4F2 genes. *Pharmacogenet Genomics* **2011**, *21*, 26-34, doi:10.1097/FPC.0b013e32834154fb.
2. Rebbeck, T.R.; Jaffe, J.M.; Walker, A.H.; Wein, A.J.; Malkowicz, S.B. Modification of clinical presentation of prostate tumors by a novel genetic variant in CYP3A4. *J Natl Cancer Inst* **1998**, *90*, 1225-1229, doi:10.1093/jnci/90.16.1225.
3. Zhou, L.P.; Yao, F.; Luan, H.; Wang, Y.L.; Dong, X.H.; Zhou, W.W.; Wang, Q.H. CYP3A4*1B polymorphism and cancer risk: a HuGE review and meta-analysis. *Tumour Biol* **2013**, *34*, 649-660, doi:10.1007/s13277-012-0592-z.
4. Nogal, A.; Coelho, A.; Catarino, R.; Morais, A.; Lobo, F.; Medeiros, R. The CYP3A4 *1B polymorphism and prostate cancer susceptibility in a Portuguese population. *Cancer Genet Cytogenet* **2007**, *177*, 149-152, doi:10.1016/j.cancergencyto.2007.06.011.
5. Pratt, V.M.; Cavallari, L.H.; Fulmer, M.L.; Gaedigk, A.; Hachad, H.; Ji, Y.; Kalman, L.V.; Ly, R.C.; Moyer, A.M.; Scott, S.A.; et al. CYP3A4 and CYP3A5 Genotyping Recommendations: A Joint Consensus Recommendation of the Association for Molecular Pathology, Clinical Pharmacogenetics Implementation Consortium, College of American Pathologists, Dutch Pharmacogenetics Working Group of the Royal Dutch Pharmacists Association, European Society for Pharmacogenomics and Personalized Therapy, and Pharmacogenomics Knowledgebase. *J Mol Diagn* **2023**, *25*, 619-629, doi:10.1016/j.jmoldx.2023.06.008.
6. Gaedigk, A.; Boone, E.C.; Turner, A.J.; van Schaik, R.H.N.; Chernova, D.; Wang, W.Y.; Broeckel, U.; Granfield, C.A.; Hodge, J.C.; Ly, R.C.; et al. Characterization of Reference Materials for CYP3A4 and CYP3A5: A (GeT-RM) Collaborative Project. *J Mol Diagn* **2023**, *25*, 655-664, doi:10.1016/j.jmoldx.2023.06.005.

7. Garsa, A.A.; McLeod, H.L.; Marsh, S. CYP3A4 and CYP3A5 genotyping by Pyrosequencing. *BMC Med Genet* **2005**, *6*, 19, doi:10.1186/1471-2350-6-19.
8. Sata, F.; Sapone, A.; Elizondo, G.; Stocker, P.; Miller, V.P.; Zheng, W.; Raunio, H.; Crespi, C.L.; Gonzalez, F.J. CYP3A4 allelic variants with amino acid substitutions in exons 7 and 12: evidence for an allelic variant with altered catalytic activity. *Clinical pharmacology and therapeutics* **2000**, *67*, 48-56, doi:10.1067/mcp.2000.104391.
9. van Schaik, R.H.; de Wildt, S.N.; Brosens, R.; van Fessem, M.; van den Anker, J.N.; Lindemans, J. The CYP3A4*3 allele: is it really rare? *Clinical chemistry* **2001**, *47*, 1104-1106.
10. Wang, A.; Yu, B.N.; Luo, C.H.; Tan, Z.R.; Zhou, G.; Wang, L.S.; Zhang, W.; Li, Z.; Liu, J.; Zhou, H.H. Ile118Val genetic polymorphism of CYP3A4 and its effects on lipid-lowering efficacy of simvastatin in Chinese hyperlipidemic patients. *Eur J Clin Pharmacol* **2005**, *60*, 843-848, doi:10.1007/s00228-004-0848-7.
11. Hsieh, K.P.; Lin, Y.Y.; Cheng, C.L.; Lai, M.L.; Lin, M.S.; Siest, J.P.; Huang, J.D. Novel mutations of CYP3A4 in Chinese. *Drug Metab Dispos* **2001**, *29*, 268-273.
12. Zhang, Y.; Wang, Z.; Wang, Y.; Jin, W.; Zhang, Z.; Jin, L.; Qian, J.; Zheng, L. CYP3A4 and CYP3A5: the crucial roles in clinical drug metabolism and the significant implications of genetic polymorphisms. *PeerJ* **2024**, *12*, e18636, doi:10.7717/peerj.18636.
13. Zhou, X.Y.; Hu, X.X.; Wang, C.C.; Lu, X.R.; Chen, Z.; Liu, Q.; Hu, G.X.; Cai, J.P. Enzymatic Activities of CYP3A4 Allelic Variants on Quinine 3-Hydroxylation In Vitro. *Frontiers in pharmacology* **2019**, *10*, 591, doi:10.3389/fphar.2019.00591.
14. Lamba, J.K.; Lin, Y.S.; Thummel, K.; Daly, A.; Watkins, P.B.; Strom, S.; Zhang, J.; Schuetz, E.G. Common allelic variants of cytochrome P4503A4 and their prevalence in different populations. *Pharmacogenetics* **2002**, *12*, 121-132, doi:10.1097/00008571-200203000-00006.
15. Maekawa, K.; Harakawa, N.; Yoshimura, T.; Kim, S.R.; Fujimura, Y.; Aohara, F.; Sai, K.; Katori, N.; Tohkin, M.; Naito, M.; et al. CYP3A4*16 and CYP3A4*18 alleles found in East Asians exhibit differential catalytic activities for seven CYP3A4 substrate drugs. *Drug Metab Dispos* **2010**, *38*, 2100-2104, doi:10.1124/dmd.110.034140.
16. Kang, Y.S.; Park, S.Y.; Yim, C.H.; Kwak, H.S.; Gajendrarao, P.; Krishnamoorthy, N.; Yun, S.C.; Lee, K.W.; Han, K.O. The CYP3A4*18 genotype in the cytochrome P450 3A4 gene, a rapid metabolizer of sex steroids, is associated with low bone mineral density. *Clinical pharmacology and therapeutics* **2009**, *85*, 312-318, doi:10.1038/clpt.2008.215.
17. Lepper, E.R.; Baker, S.D.; Permenter, M.; Ries, N.; van Schaik, R.H.; Schenk, P.W.; Price, D.K.; Ahn, D.; Smith, N.F.; Cusatis, G.; et al. Effect of common CYP3A4 and CYP3A5 variants on the pharmacokinetics of the cytochrome P450 3A phenotyping probe midazolam in cancer patients. *Clinical cancer research : an official journal of the American Association for Cancer Research* **2005**, *11*, 7398-7404, doi:10.1158/1078-0432.CCR-05-0520.
18. Dai, D.; Tang, J.; Rose, R.; Hodgson, E.; Bienstock, R.J.; Mohrenweiser, H.W.; Goldstein, J.A. Identification of variants of CYP3A4 and characterization of their abilities to metabolize testosterone and chlorpyrifos. *The Journal of pharmacology and experimental therapeutics* **2001**, *299*, 825-831.
19. Du, J.; Xing, Q.; Xu, L.; Xu, M.; Shu, A.; Shi, Y.; Yu, L.; Zhang, A.; Wang, L.; Wang, H.; et al. Systematic screening for polymorphisms in the CYP3A4 gene in the Chinese population. *Pharmacogenomics* **2006**, *7*, 831-841, doi:10.2217/14622416.7.6.831.
